# Evidence based disease control methods in potato production: a systematic map protocol

**DOI:** 10.1186/s13750-022-00259-x

**Published:** 2022-02-18

**Authors:** Elisa Vilvert, Linnea Stridh, Björn Andersson, Åke Olson, Louise Aldén, Anna Berlin

**Affiliations:** 1 Dept. Forest Mycology and Plant Pathology, Swedish University of Agricultural Science, Box 7026, 75007 Uppsala, Sweden; 2 Dept. Plant Protection Biology, Swedish University of Agricultural Science, PO Box 102, 23053 Alnarp, Sweden; 3 Sveriges Stärkelseproducenter Förening, Degebergavägen 60-20, 29191 Kristianstad, Sweden; 4 Swedish Board of Agriculture, Plant Protection Centre, Elevenborsgvägen 4, 23456 Alnarp, Sweden

**Keywords:** *Solanum tuberosum*, Plant diseases, Plant protection, Starch potato, Table potato

## Abstract

**Background:**

Several challenges, e.g. global trade, population growth, and climate change create future challenges for food production and food safety. In order to meet this, we need to secure and increase agricultural production with minimal environmental impact. Potato (*Solanum tuberosum*) ranks as one of the world’s most important crops for human consumption. While potato production and consumption have decreased in Europe and North America, global production has grown in the last decades due to the expansion of potato consumption in Asia. Potato is vulnerable to a wide range of pathogenic organisms, all of which can cause severe quality and yield losses. As a consequence, potato production is highly reliant on pesticide use, and this has a negative effect on the sustainability of the crop. To mitigate these problems, effective and evidence based crop protection recommendations need to be provided to growers.

**Methods and output:**

The overarching aim of this project is to support the development of better methods of integrated pest management (IPM), as well as to identify alternative control methods for potato diseases to contribute to effective plant protection solutions and a more sustainable potato production. The specific objective of this systematic map is to provide a worldwide overview of plant disease protection measures available for potato production. All methods to control diseases within different cropping systems will be considered, such as pesticide application, biological control methods, resistant cultivars as well as disease support systems and tools for diagnosis. The systematic map will be presented as a searchable database where the volume and main characteristics of the relevant scientific literature will be described. We will identify evidence clusters and knowledge gaps in potato disease management and identify future research areas, and in this way contribute to new and innovative solutions. The map will provide important information and support for researchers and stakeholders, in particular authorities and advisory organizations. It will also help to select topics for future systematic reviews and meta-studies within potato research.

**Supplementary Information:**

The online version contains supplementary material available at 10.1186/s13750-022-00259-x.

## Background

Potato (*Solanum tuberosum*) ranks as the world’s third most important crop for human consumption, after rice and wheat. Throughout its history, the potato has been contributing to food security and poverty eradication [1, 2]. From the mid-eighteenth to mid-nineteenth century, potato production was the main factor in feeding the increasing European population. Today, potato still contributes to global food security [1] through characteristics such as its ability to provide high yields in a short time, low land demand, and its adaptability to a wide range of environments [1, 3].

The domestication of potato took place around 8000 years ago in the vicinity of Lake Titicaca located in the Andean region on the border between what today is Peru and Bolivia [1]. The potato was introduced in Europe in the sixteenth century after the Spanish conquest of Peru (1532–1572) and subsequently spread around the world. In Europe, the potato was first primarily grown in botanical gardens and it was not until in the 1770s when most of Europe was devasted by famines, that potato started to be recognized as an important food crop [1, 4]. In the 1840s the potato crops failed as a result of late blight (*Phytophthora infestans*), which devasted potato fields across Europe. This led to extreme famine, especially in Ireland (1845–1848) where failing potato crops caused the deaths of one million people and a wave of emigration. This led to the introduction of the practice of fungicide treatments and the development of new disease resistant potato varieties which since then have been the base of successful potato production in Europe [4, 5].

**Fig. 1:**
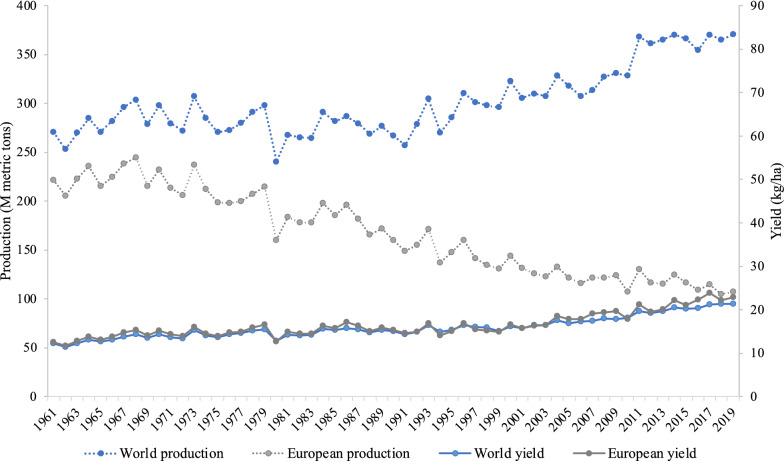
Comparison between the world and European potato production (million metric tons) and the world and European yield (kg/ha) between 1961 and 2019

In the past decades, the consumption of fresh potatoes has decreased in Europe and North America due to the increasing popularity of other staple carbohydrates such as pasta, rice, and bulgur [6]. As a result, the European potato production has been reduced by half in the last 60 years, from 221.8 million metric tons in 1961 to 107.3 million metric tons in 2019 [7]. Concurrently, the potato yield per hectare has significantly increased during this period due to the use of good crop management and successful breeding efforts [3, 8]. Contrary to the decreasing trend in potato production and consumption observed in Europe and USA, the global potato production has shown an expansion in the last decades (Fig. 1). This increase is mainly driven by the increased potato production and consumption in China and India [1]. China is currently the world’s largest potato producer accounting for 25% of total global production followed by India (14%) and Russia (6%) [7]. In addition, an increase in potato production is taking place in many low-income countries where potato is playing an increasingly important role in food security [2].

Potato is produced for different purposes: fresh consumption (baked, boiled, or fried); processed food products (frozen French fries, potato crisps, dehydrated potato); potato starch for industry (food additives, pharmaceutical, textile, wood, and paper industries); seed potatoes; and, to a lesser extent, for animal feed [4]. Thousands of different potato cultivars are available with differences in size, shape, color, texture, cooking characteristics, taste, starch content, and disease resistance [4]. The decision on which cultivar to grow is based not only on the local climatic conditions and agronomic characteristics, but also on the market purpose of the potato crop.

Potato is susceptible to a wide range of pathogenic organisms, which all can cause severe quality and yield losses. The diseases caused by these pathogens result in significant losses by affecting the potato quality during cultivation, storage and processing [3]. Some of the most important diseases worldwide are late blight (*Phytophthora infestans*), early blight (*Alternaria solani*), stem canker (*Rhizoctonia solani*), potato wart (*Synchytrium endobioticum*), powdery scab (*Spongospora subterranea*), bacterial wilt (*Ralstonia solanacearum*), black leg (*Pectobacterium* spp.), potato virus Y (PVY), potato leaf roll virus (PLRV) and yellow potato cyst nematode (*Globodera rostochiensis*) [9–11]. However, the relative importance of a disease vary between production markets and between growing regions according the pathogen distribution and the local climatic conditions. The oomycete *P. infestans*, causing late blight and tuber blight, is still one of the main biotic constraints of potato production worldwide due to it aggressiveness and genetic variability [9]. Under optimal conditions, late blight can cause severe crop damage within a week. Fungicide use is still the most common control management strategy because of a dominance of varieties with low or moderate resistance to late blight due to low marketability and acceptance of resistant cultivars [9, 12]. As a direct consequence of late blight, potato is one of the most fungicide dependent crops, and in many countries potato receives the largest amounts of pesticides per hectare of all crops grown [13]. In Sweden, potato production occupies only 0.9% of the arable land area, while 21% of all fungicides used in Swedish agriculture are applied in potato crops [8]. As an example, data from 2017 shows that potato crops in Sweden received 2.0 kg fungicides per ha, while only 0.1 kg per ha^1^ was used in cereals [14].

Increasing concerns about the health and environmental consequences of pesticide use have led to strict regulations on pesticide use and a decrease in the number of fungicides approved for potato disease management in many countries [9]. Presently, integrated pest management (IPM) is the most recommended management strategy in plant protection. The EU directive on sustainable use of pesticides (2009/128/EC) which legislate the application of IPM, emphasizes the production of healthy crops with the least impact on the agro-ecosystem [15]. IPM strategies require the combination of several approaches: preventive measures such as prediction of a disease outbreak, good cultural practices (such as the use of healthy seeds, resistant cultivars, crop rotation, etc.), and the application of pesticides according to the need determined via disease monitoring (use of diagnostic tools) or forecast [5, 9]. In the case of potato, a possible way of obtaining a more sustainable production is a combination of host resistance and the implementation of need-based fungicide use. Investment in breeding programs for the development of varieties with better resistance to *P. infestans* (a longstanding potato breeding objective), and other pathogens (fungi, virus, nematodes, and bacteria) offers improved sustainability in the production of potato by reducing the need for pesticides. Development of biocontrol agents, plant resistance inducers (PRIs), and other low-risk compounds is very active and considered to grow substantially in the coming decade. However, there is still little confirmed success from the use of this type of control measures under field conditions, indicating the need for more field-based research [3]. Moreover, the use of sustainable management practices as healthy and certificated seed, elimination of volunteers, and crop rotation, for example, should be enforced to avoid or reduce pathogen survival, dispersal, and reproduction [9]. However, disease management recommendations may vary and should be adapted to the specific production region and the environmental conditions [16].

Disease diagnostic tools and decision support systems (DSS) are important elements of IPM strategies since they are both used for assisting farmers and advisors in their decisions about crop protection methods. Diagnostic tools are essential to detect and correctly identify diseases in an early stage of development and in this way support timely control measures [17]. These tools comprise of direct identification through visual assessments (by eye or with the aid of a microscope), immunological diagnostic methods (enzyme immunoassays such as Enzyme Linked Immunosorbent Assays—ELISA), and molecular diagnostic tools based on a range of PCR and sequencing methods. In addition, the use of remote sensing or spore trapping are useful to monitor larger areas that could not be adequately monitored by a direct inspection [17]. Sometimes visual identification of disease symptoms on plants can be difficult [17], and other diagnostic tools are often required for a more reliable diagnosis.

DSS are interactive tools, developed to manage complex problems under uncertain conditions through the use of data and simulation modeling. DSS for plant protection are developed to predict disease epidemics, and are often based on known dependencies between weather factors such as air humidity and temperature and disease development. The output from DSS’s varies from simple risk evaluation to precise advice on the most appropriate control method [18, 19]. If there is a need for fungicide application, some DSS can indicate the appropriate application time, the ideal active substance, and dosage. In this way, optimization of fungicide use can be achieved, often resulting in a reduction of costs and minimized environmental pollution [19, 20].

### Topic identification and stakeholder engagement

Potato production requires high amounts of pesticides to limit the negative impact of potato diseases due to aggressive plant pathogens in combination with the use of susceptible cultivars. Efficient management of this complex system requires the implementation of all available knowledge. The primary goal of this project is to support the development of better methods of IPM, as well as to identify alternative control methods for potato diseases, which will contribute to effective plant protection solutions and more sustainable potato production.

The topic of this systematic map, as well as the formulation of the primary and secondary questions, were actively discussed with representatives with interest in potato production from academia, advisory service, and industry. To ensure the relevance of the primary and secondary questions, the group was also consulted in the development of the eligible criteria.

## Objective of the systematic map

The objective of this systematic map is to provide a worldwide overview of evidence based plant disease control measures in potato production. The systematic map will describe the volume and main characteristics of the scientific literature and identify evidence clusters and knowledge gaps in potato disease management and identify future research areas, and in this way contributing to new and innovative solutions.

### Primary question of the systematic map

What is the evidence base of plant disease protection measures and strategies available for potato production?

### Secondary questions

Two secondary questions will complement the analysis to ensure that all possible tools for disease control will be identified.

a) *Which decision support systems exist for disease management in potato production?*

b) *What are the available disease diagnostic methods?*

### Components of the primary question (PICO)

*Population* Potato (*Solanum tuberosum*).

*Intervention* Any measure to control plant diseases, both direct and indirect interventions including disease management support system and diagnostic methods.

*Comparator* Comparison between intervention and no intervention (control) or between different interventions.

*Outcomes* Yield or outcome measured as yield per area, disease suppression or reduction measured as incidence or severity on plant parts, or increase in quality.

## Methods

The method follows the Collaboration for Environmental Evidence Guidelines and Standards for Evidence Synthesis in Environmental Management [21] and conforms to the ROSES reporting standard [22] (see Additional file 1). This systematic map protocol builds on our previously published protocol [15] used to identify disease management methods in field crops [23]. In this protocol, we have widened the scope to be applicable for potato production worldwide as well as updated the search string and the screening criteria.

### Scoping

The search strategy is designed to retrieve a broad range of articles covering all types of disease control methods in potato production worldwide. The search string was developed and reviewed in discussions with both researchers and persons actively working in the field of plant protection. As a scoping exercise, we conducted test searches with different words in in five scientific bibliographic databases (Web of Science core collection, Biosis Citation Index, CABI, Scopus and Agris). The search string which returned the highest number of articles capturing relevant publications was selected. To test the robustness of the search string, all searches were performed by two independent persons and a substantial agreement (99.9%) was achieved (see Additional file 2).

### Searching for publications

All searches will be performed in English, and only studies published or translated to English will be included in this systematic map. A time-span restriction for research published from 2000 until 2021 will be applied in all searches.

### Search string

The search string was structured in four thematic blocks: crop, disease-causing organism, plant disease control, and outcome. Search terms were truncated and a (*) was added at the end of the root world to include all alternative forms of the word to allow for inclusion of other spelling or hyphenation of a word. Quotation marks were placed around multiple words terms to search for exact phrases. All search words within a thematic block were combined using “OR” and the blocks were then combined using “AND”. The search string used is presented in Table 1.

**Table 1:** Search string for the four thematic blocks

Thematic block	Search string
Crop	Potato OR “Solanum tuberosum”
Disease causing organisms	Fung* OR oomycete* OR nematod* OR bacter* OR virus* OR viral OR viroid* OR pathogen*
Plant disease control	“Plant protection” OR “control strateg*” OR “risk management” OR “biological control” OR “disease control” OR IPM OR “integrated pest management” OR pesticid* OR fungicid* OR herbicid* OR insecticide* OR “plant defen*” OR resistance OR “disease develop*”
Outcome	“Disease incidence” OR “disease severity” OR “plant health” OR yield* OR qualit* OR harvest OR produc* OR “pathogen reduction”

### Bibliographic databases

The searches will be conducted in five scientific bibliographic databases (Table 2). The first three databases (Web of Science core collection, Biosis Citation Index, and CABI) will be accessed through the Swedish University of Agricultural Sciences (SLU) subscription at Web of Science (v.5.30) and the remaining two (Scopus and AGRIS) will be accessed directly through their websites. A shorter search string was created for AGRIS to adapt to the search function of the database. The number of articles captured, the date of search, database, and platform name will be recorded. In the scoping exercise, the number of hits ranged between 754 and to 9102 between the different databases (Table 2).

**Table 2:** Search results in the scoping exercise for the search string developed in this protocol in the five scientific bibliographic databases (2000–2021)

Database	Date of search	Number of hits
Web of Science core collection	2021-08-18	3 792
Biosis Citation Index^a^	2021-08-18	2 538
CABI: CAB Abstract and Global Health	2021-08-18	9 102
Scopus	2021-08-18	3 601
Agris^b^	2021-08-18	754

^a^ (2009–2021)

^b^ A shorter search string will be used: (Fung* OR oomycete* OR nematod* OR bacter* OR virus* OR viral OR viroid* OR pathogen*) AND (potato OR “*Solanum tuberosum*”)

The results of the search from each selected databases will be imported into separate EndNote X9 library files. The files for the different databases will then be combined into one single file. Using the automatic duplicate identifier function in EndNote X9 all duplicates will be identified and removed after manual inspection and the number of removed publications will be recorded.

### Specialist search for grey literature

A search for grey literature will be performed using three approaches: preprint archives, regional and cross-national organizations, and Google Scholar. For the two first searches, a shorter search string will be used comprising the scientific name (“*Solanum tuberosum*”) and the common name of the crop (Potato). First, databases for preprint archives such as bioRxiv (http://www.biorxiv.org), PeerJ (http://www.peerj.org), and arXiv (http://www.arxiv.org) will be searched to identify prepublished original studies. Secondly, regional and cross-national organization’s webpages indicated as relevant by stakeholders and authors will be searched to identify studies not published in scientific journals.

Webpages of transnational organizations with activities within plant protection:

CGIAR, Consultative Group for International Agricultural Research (https://www.cgiar.org).

CIP, The National Potato Center (https://cipotato.org).

EFSA, European Food Safety Authority (http://www.efsa.europ a.eu).

EPPO, European and Mediterranean Plant Protection Organization (https://www.eppo.int).

European Crop Protection Association (https://www.ecpa.eu).

FAO, Food and Agricultural Organization of the United Nation (http://www.fao.org).

IPPC, International Plant Protection Convention (https://www.ippc.int/en/).

NAPPO, North American Plant Protection Organization (http://www.nappo.org).

Finally, the limited search string developed for the AGRIS database will be used for search in Google Scholar using the Publish or Perish software [24] and the 1000 first records will be downloaded and included in the EndNote library.

### Article screening

All articles will initially be screened in two stages, (1) title and abstract and (2) full text level. At each step, the inclusion or exclusion of articles from the map will be based on agreed eligibility criteria. If the relevance of a publication is unclear at stage 1, it will be included and assessed at full text level. All articles excluded at the title and abstract level will be recorded together in one file. Articles excluded at full text level will be recorded in a separate file including motivation for exclusion. These lists will be supplied as additional information in an excel file in the report.

To ensure consistency and high quality in the screening process, an initial test based on a sub-set of 80 articles retrieved by the searches will be checked against the eligible criteria at title, abstract and full text level by four members with expertise in the subject independently. A kappa test will be used to determine agreement at both levels, with a score of 0.6 or above indicating substantial agreement [25]. Disagreement will be discussed and the eligible criteria will be clarified if needed.

The full screening process will primarily be carried out by one core reviewer. At each stage of the screening (title and abstract level and full text level), a random 25% of articles will be selected for screening by at least one additional reviewer. Based on this, a kappa test will be used to determine the proportional agreement between the reviewers, and an agreement with a score of 0.6 or above is expected, indicating substantial agreement [25]. Any disagreements will be discussed and if different opinions remain after discussion, the article will be included in the map.

Reviewers that are authors of relevant articles will not be included in the decision connected to the inclusion and study validity assessment of their articles.

### Eligibility criteria

Publications fulfilling the following eligibility criteria will be included in the systematic map:

#### Eligible population

Studies including original research about potato *Solanum tuberosum*) in field trials, in pot trials in soil or post-harvest storage as well as articles reporting about any disease support system or tool for potato disease diagnostic method published between 2000–2021.

#### Eligible intervention

Studies of any disease management intervention, independently or in combination, including but not limited to crop rotation, resistant cultivars, cultivar mixtures, plowing, no-tillage, biological control, bio fungicide, and pesticide applications. Only studies including active substances not banned for use against plant diseases in the EU will be included. For this, a list of active substances banned for use in the EU will be retrieved from the European Commission online database (https://ec.europa.eu/food/plant/pesticides) at the start of the articles screening process. The decision in using the EU list of pesticide active substances is based in the fact that EU currently has one of the most comprehensive and protective pesticide regulation allowing the market of substances that do not result in harm to human, animals and the environment [26].

#### Eligible outcome

Studies reporting any type of effect of disease control interventions that are measured in productivity in terms of the total harvest, yield per area, or relevant crop quality measures e.g., decrease in toxin levels, plant health status, or reduced disease symptoms. Disease reduction can also be included as a proxy for potential yield increase or increase in crop quality.

#### Eligible study type(s)

Three types of studies will be considered eligible for this map:

(i) Original research based on relevant experimental study design including, but not limited to, before and after studies (BA), before and after control impact studies (BACI), randomized control trials (RCT) randomized split block trials (RSBT), and exposure versus no exposures/control impact (CI). Articles and reports not including original data and/or limited statistical evaluation of results will not be included.
(ii) Studies reporting any type of DSS or a component of a DSS (i.e., simulation models). The inclusion criteria for these studies will focus on population and if it was designed to assist in decision making for disease control.
(iii) Studies reporting disease diagnostic tools or methods. The diagnosis method should have been tested and validated on samples produced in the greenhouse or collected from fields.

In addition, books or book chapters, review articles, and reports with no relevant study design will be recorded in a separate folder named “Book, reviews and reports”. The reference lists of these studies will be screened, and if relevant research articles are identified that meet the eligibility criteria, these articles will be included in the systematic map database.

Articles that are not accessible as full text online (through the SLU subscription or as open access) will be excluded.

### Study validity assessment

A basic quality assessment and identification of the design of the experiment will be performed when evaluating the relevance of the study and if they are eligible to be included in the map. This information will be included in the coding of each study and a brief description of the type of study will be included as “free text” when considered informative. No further study validity assessment or critical appraisal of the included studies will be performed since the intention of this map is to provide an overview of the available literature about disease control methods, DSS, and diagnostic tools in potato production.

### Data coding strategy

Standardized descriptive metadata and descriptive study information from all studies meeting the eligibility criteria will be stored in an Excel file, which will form the systematic map database. Data from each study will be coded as described in Table 3. First, a sub-set of included studies will be coded by at least two reviewers, and if any discrepancy, these will be discussed. The complete data coding will be performed by one core reviewer.

**Table 3:** Descriptive information retrieved for all studies included in the systematic map

Code	Description
Bibliographic information	Unique reference ID, Reference type, Year of publication, Authors, Title, Journal, Volume, Page number, URL, or DOI
Location(s) of study	Country (and region when relevant)
Disease(s)	Common name of the disease(s)
Pathogen type(s)	Fungi, bacteria, viruses, viroids, virus-like organisms, phytoplasmas, protozoa, oomycetes, nematodes, or parasitic plants
Disease-causing organism(s)	Scientific names of organisms causing the diseases
Study type(s)	i) Any type of experimental study designs used in field or pots with soil that can be statistically evaluated: before and after studies (BA), before and after control impact studies (BACI), randomized control trials (RCT) randomized split block trials (RSBT) and exposure versus no exposures/control impact (CI) ii) Studies reporting a Disease Support System iii) Studies reporting disease diagnosis method(s)
Management(s)	Any type of disease control intervention or management: application of any type of pesticide or biological control agent, any type of seed management, cultivar resistance, or cultivar mixtures, etc. If a management agricultural practice was evaluated to disease control, then it was also included: any type of agricultural practices such as crop rotation, intercrop systems, any type of soil management, fertilization, plant density, time of sowing, and harvest techniques
Diseased part(s):	Tuber, roots, leaf, stem, flower
Plant stage(s)	Seed potato, plantlet, adult, mature, or post-harvest
Outcome(s)	Any effect of disease control measured: yield, crop quality, plant health, disease incidence, disease severity
GMO	Yes or No, if yes: specify which type of trait the included cultivar(s) have been given
Decision support system(s)	Characteristics of DSS used for assisting farmers in decision-making for disease control
Diagnostic tool(s)	Type of diagnostic tool including, but not limited to the ocular, immunological, or molecular method for identification of potato disease

Studies included in the category “Book, reviews and reports” will be sub-divided in different groups, including but not restricted to: (i) peer-reviewed review articles, (ii) books or book chapters, and (iii) conference contributions and reports (with no relevant study design or not statistically evaluated).

### Study mapping and presentation

The final report will narratively describe the extracted metadata as well as describe the review process, the amount and nature of available studies describing plant disease control methods, DSS, and diagnostic tools available in potato production worldwide in text, tables, and figures. The studies will be grouped and presented in different categories, i.e., pathogen type and disease or intervention. Included studies and their metadata will be presented in a searchable Excel database that will be made available as an additional file with the published systematic map report. Identified knowledge clusters, gaps, and excesses will be presented and discussed. Based on the findings, the report will also include a critical analysis of the findings and their implications for research as well as policy-makers and management.

## Supplementary Information

**Additional file 1. ** ROSES for Systematic Map Protocols Version 1.0.

**Additional file 2. **Description of the scoping exercise and the database search results.

## Data Availability

All data generated or analyzed during this study are included in this published article and its supplementary information files.
