# Supplementary material for: Evidence based disease control methods in potato production: a systematic map protocol
Source: Environ Evid. 2022 Feb 18;11:6. doi: 10.1186/s13750-022-00259-x (PMC11378795; doi:10.1186/s13750-022-00259-x)
Supplement: Supplementary file 2 — Additional file 2. Description of the scoping exercise and the database search results. [file 13750_2022_259_MOESM2_ESM.docx]

**Summary of scoping searches**

Two search strings were tested, the search string developed and presented in Berlin et al 2018 (Table a1 below) was compared with an updated version (Table 1 in this systematic map protocol).

**Table a1. Search string for the four thematic blocks**

| **Thematic block** | **Search string** |
| --- | --- |
| Crop | Potato OR “Solanum tuberosum” |
| Disease causing organisms | Fung* OR oomycete* OR nematod* OR bacter* OR virus* OR viral OR viroid* OR pathogen* |
| Plant disease control | “Disease incidence” OR “disease severity” OR “plant protection” OR “control strateg*” OR “risk management” OR “biological control” OR “disease control” OR IPM OR “integrated pest management” OR “plant defen*” OR resistance OR “disease develop*” |
| Outcome | “Plant health” OR yield* OR qualit* OR harvest OR produc* OR “pathogen reduction” |

All test searches were performed by two independent reviewers and conducted the 18^th^ of August 2021. For all searches, a time restriction for 2000 – 2021 was applied (i.e., 2000-01-01 to 2021-08-18). The updated search string could identify a larger number of articles (Table a2) than the one published in Berlin et al (2018) and was thus selected to use for the systematic map.

**Table a2. Number of articles identified by two independent reviewers**

|  | **Berlin *et al* 2018** | | **Updated search string** | |
| --- | --- | --- | --- | --- |
| **Database** | Reviewer 1 | Reviewer 2 | Reviewer 1 | Reviewer 2 |
| Web of Science core collection | 3 351 | 3 351 | 3 792 | 3 792 |
| Biosis Citation Index ^a^ | 1 889 | 1 889 | 2 538 | 2 538 |
| CABI: CAB Abstract and Global Health | 7 510 | 7 510 | 9 102 | 9 102 |
| Scopus | 3 157 | 3 157 | 3 601 | 3 601 |
| Agris ^b^ | 754 | 754 | 754 | 754 |

^a^ (2009-2021)

^b^ A shorter string was used in for both old and new searches: (Fung* OR oomycete* OR nematod* OR bacter* OR virus* OR viral OR viroid* OR pathogen*) AND (potato OR “*Solanum tuberosum*”)

**Details of search string for the different searches:**

**Web of Science core collection, BIOSIS Citation index** **and** **CABI: CAB Abstract and Global Health (Berlin et al 2018)**

The four blocks were first searched independently and then combined with “AND”.

TS=(potato OR "Solanum tuberosum")

TS=(fung* OR oomycete* OR nematod* OR bacter* OR virus* OR viral OR viroid* OR pathogen*)

TS=(“disease incidence” OR “disease severity” OR “plant protection” OR “control strateg*” OR “risk management” OR “biological control” OR “disease control” OR IPM OR “integrated pest management” OR “plant defen*” OR resistance OR “disease develop*”)

TS=(“plant health” OR yield* OR qualit* OR harvest OR produc* OR “pathogen reduction”)

**Web of Science core collection, BIOSIS Citation index** **and** **CABI: CAB Abstract and Global Health (Updated search string)**

The four blocks were first searched independently and then combined with “AND”

TS=(potato OR "Solanum tuberosum")

TS=( fung* OR oomycete* OR nematod* OR bacter* OR virus* OR viral OR viroid* OR pathogen*)

TS=("plant protection" OR "control strateg*" OR "risk management" OR "biological control" OR "disease control" OR IPM OR "integrated pest management" OR "plant defen*" OR resistance OR "disease develop*" OR pesticid* OR fungicid* OR herbicid* OR insecticid*)

TS=("disease incidence" OR "disease severity" OR "plant health" OR yield* OR qualit* OR harvest OR produc* OR "pathogen reduction")

**Scopus (Berlin et al 2018)**

TITLE-ABS-KEY (potato OR "Solanum tuberosum" AND fung* OR oomycete* OR nematod* OR bacter* OR virus* OR viral OR viroid* OR pathogen* AND "disease develop*" OR "disease incidence" OR "plant protection" OR "control strateg*" OR "risk management" OR "biological control" OR "disease control" OR IPM OR "integrated pest management" OR "plant defen*" OR resistance OR "disease severity" AND "plant health" OR yield* OR qualit* OR harvest OR produc* OR "pathogen reduction") AND PUBYEAR > 1999 AND PUBYEAR < 2022

**Scopus (Updated version)**

TITLE-ABS-KEY (potato OR "Solanum tuberosum" AND fung* OR oomycete* OR nematod* OR bacter* OR virus* OR viral OR viroid* OR pathogen* AND "plant protection" OR "control strateg*" OR "risk management" OR "biological control" OR "disease control" OR IPM OR "integrated pest management" OR "plant defen*" OR resistance OR "disease develop*" OR pesticid* OR fungicid* OR herbicid* OR insecticid* AND "disease incidence" OR "disease severity" OR "plant health" OR yield* OR qualit* OR harvest OR produc* OR "pathogen reduction") AND PUBYEAR > 1999 AND PUBYEAR < 2022

**AGRIS (same search string for Berlin et al 2018 and updated version)**

(potato OR “Solanum tuberosum”) AND (Fung* OR oomycete* OR nematod* OR bacter* OR virus* OR viral OR viroid* OR pathogen*)
